# Sedentary and Trained Older Men Have Distinct Circulating Exosomal microRNA Profiles at Baseline and in Response to Acute Exercise

**DOI:** 10.3389/fphys.2020.00605

**Published:** 2020-06-10

**Authors:** Venugopalan D. Nair, Yongchao Ge, Side Li, Hanna Pincas, Nimisha Jain, Nitish Seenarine, Mary Anne S. Amper, Bret H. Goodpaster, Martin J. Walsh, Paul M. Coen, Stuart C. Sealfon

**Affiliations:** ^1^Department of Neurology, Center for Advanced Research on Diagnostic Assays, Icahn School of Medicine at Mount Sinai, New York, NY, United States; ^2^Department of Pharmacological Sciences, Icahn School of Medicine at Mount Sinai, New York, NY, United States; ^3^Translational Research Institute, AdventHealth, Orlando, FL, United States

**Keywords:** Acute aerobic exercise, regular endurance training, aging, plasma exosomes, microRNA profiling, insulin growth factor-1 signaling

## Abstract

Exercise has multi-systemic benefits and attenuates the physiological impairments associated with aging. Emerging evidence suggests that circulating exosomes mediate some of the beneficial effects of exercise via the transfer of microRNAs between tissues. However, the impact of regular exercise and acute exercise on circulating exosomal microRNAs (exomiRs) in older populations remains unknown. In the present study, we analyzed circulating exomiR expression in endurance-trained elderly men (*n* = 5) and age-matched sedentary males (*n* = 5) at baseline (Pre), immediately after a forty minute bout of aerobic exercise on a cycle ergometer (Post), and three hours after this acute exercise (3hPost). Following the isolation and enrichment of exosomes from plasma, exosome-enriched preparations were characterized and exomiR levels were determined by sequencing. The effect of regular exercise on circulating exomiRs was assessed by comparing the baseline expression levels in the trained and sedentary groups. The effect of acute exercise was determined by comparing baseline and post-training expression levels in each group. Regular exercise resulted in significantly increased baseline expression of three exomiRs (miR-486-5p, miR-215-5p, miR-941) and decreased expression of one exomiR (miR-151b). Acute exercise altered circulating exomiR expression in both groups. However, exomiRs regulated by acute exercise in the trained group (7 miRNAs at Post and 8 at 3hPost) were distinct from those in the sedentary group (9 at Post and 4 at 3hPost). Pathway analysis prediction and reported target validation experiments revealed that the majority of exercise-regulated exomiRs are targeting genes that are related to IGF-1 signaling, a pathway involved in exercise-induced muscle and cardiac hypertrophy. The immediately post-acute exercise exomiR signature in the trained group correlates with activation of IGF-1 signaling, whereas in the sedentary group it is associated with inhibition of IGF-1 signaling. While further validation is needed, including measurements of IGF-1/IGF-1 signaling in blood or skeletal muscle, our results suggest that training status may counteract age-related anabolic resistance by modulating circulating exomiR profiles both at baseline and in response to acute exercise.

## Introduction

Aging is associated with loss of skeletal muscle mass and function, physiological alterations of the cardiorespiratory system, weakening of the immune system, and impaired cognitive function (Garatachea et al., 2015; Weyand and Goronzy, 2016). While a sedentary lifestyle may accelerate the hallmarks of aging, regular exercise can lessen those multisystem age-related declines, thereby decreasing the risk of cardiovascular diseases, cancers, and metabolic disorders (Febbraio, 2017; Harridge and Lazarus, 2017; Howden et al., 2018; Chowdhury et al., 2019). Major long-term adaptations to exercise training include improved cardiovascular and cardiorespiratory fitness, enhancement of skeletal muscle blood flow and oxidative capacities (via an increased density of capillaries and of mitochondria, respectively) (Rivera-Brown and Frontera, 2012), and muscle hypertrophy (Konopka and Harber, 2014). Even a single bout of exercise can induce several health benefits in skeletal muscle, such as enhanced insulin sensitivity, increased secretion of the anti-inflammatory cytokine interleukin-6, and stimulation of immune cells (Garatachea et al., 2015; Sellami et al., 2018).

While the molecular mechanisms underlying the health benefits of exercise remain unclear, accumulating evidence suggests the importance of tissue crosstalk via the release of molecules into the bloodstream (i.e., circulation). Many of these molecules are packaged into extracellular vesicles (EVs) such as microvesicles and exosomes (Whitham et al., 2018). Exosomes are nano-sized (30–150 nm) EVs that transport proteins and various RNA species, notably miRNAs (Kowal et al., 2014). Exosomes are found in most body fluids, including plasma, urine, and saliva. There is growing evidence that exercise leads to the release of exosomes by skeletal muscle and other tissues into circulation, suggesting that these EVs act as mediators of the systemic adaptations to exercise (Chaturvedi et al., 2015; Fruhbeis et al., 2015; Safdar and Tarnopolsky, 2018; Whitham et al., 2018).

Among the molecules transported by exosomes, microRNAs (miRNAs) are small non-coding RNAs that mediate post-transcriptional gene silencing. miRNAs are important regulators of gene expression (Selbach et al., 2008; O’Brien et al., 2018). Extracellular (or circulating) miRNAs consist of two populations: those associated with vesicles such as exosomes, and those bound to proteins like AGO2 (O’Brien et al., 2018). The majority of previous studies on circulating miRNA responses to exercise measured changes in vesicle-free extracellular miRNAs in either plasma or serum (Baggish et al., 2011, 2014; Aoi et al., 2013; Mooren et al., 2014; Nielsen et al., 2014; Russell and Lamon, 2015; Xu et al., 2015; D’Souza et al., 2017; Shah et al., 2017; Horak et al., 2018; Barber et al., 2019). Those studies have suggested that circulating miRNAs may mediate transient metabolic changes and adaptive responses induced by exercise. However, recent reports involving healthy young men suggest that the exosomal fraction is distinctively modulated by exercise and may play an important role in exercise-induced systemic adaptations (Guescini et al., 2015; D’Souza et al., 2018; Lovett et al., 2018; Hou et al., 2019).

Aging has been associated with alterations in intramuscular and circulating miRNA profiles (Drummond et al., 2011; Noren Hooten et al., 2013; Lai et al., 2014; Olivieri et al., 2014b; Zhang et al., 2015). However, the effect of exercise on the circulating exosomal miRNA (exomiR) expression profiles of aging individuals has not been investigated. Here, we studied the impact of long-term exercise training and acute endurance exercise on circulating exosomal miRNAs using a comprehensive small RNA sequencing-based approach. The results indicate that long-term exercise affects basal (resting) circulating exomiR profiles and demonstrate that acute exercise generates distinct patterns of exomiR profiles specific to trained and sedentary groups.

## Materials and Methods

### Subjects

Older men (>65 years old) were recruited from the Orlando, FL, United States area. Participants were in good general health, defined as having no chronic medical conditions (i.e., type I or II diabetes), no contraindications for exercise, stable weight for the last 6 months, and controlled hypertension (<150 mmHg systolic, <90 mmHg diastolic). Participants (*n* = 10) were assigned to one of two groups based on their physical activity levels: 5 older trained and 5 older sedentary. Subject characteristics are summarized in Table 1. Trained subjects were engaged in endurance activities (running, cycling) for at least 5 years without an extensive layoff (>6 months) due to injury. Sedentary individuals completed ≤1 continuous exercise bout per week. Each participant provided written informed consent before completing any data collection procedures. The study was approved by the Florida Hospital, AdventHealth Orlando (Orlando, FL, United States) Institutional Review Board (IRBNet #554559) and performed in compliance with the Declaration of Helsinki.

**TABLE 1:** Baseline subject characteristics.

**Clinical variable**	**Trained (*n* = 5)**	**Sedentary (*n* = 5)**	T-test*
Age (yrs)	68.2 ± 1.6	70.4 ± 1.4	0.331
Weight (kg)	77.1 ± 7.9	89.6 ± 7.5	0.284
BMI (kg/m^2^)	26.1 ± 2.4	28.7 ± 1.8	0.411
Total Fat Mass (kg)	20.2 ± 5.2	31.8 ± 4.8	0.140
SMI (ALM/height^2^)	8.5 ± 0.5	8.2 ± 0.5	0.683
Total Lean Mass (kg)	55.0 ± 3.3	55.0 ± 3.2	1
Leg Lean Mass (kg)	9.0 ± 0.6	9.5 ± 0.7	0.602
VO_2_ max (ml/kgBW/min)	34.4 ± 1.1	21.7 ± 1.2	< 0.0001
Daily Average Steps (steps/24 h)	6386 ± 410	4916 ± 1370	0.354

Values are expressed as mean +/− SEM. *Unpaired *t* test; BMI, body mass index; SAT, subcutaneous adipose tissue; SMI, skeletal muscle index; ALM, appendicular skeletal muscle mass; Total Lean Mass = Total Weight − Fat Mass; Muscle Volume, volume of muscle tissue; VO_2_ max, maximal oxygen uptake (cardiorespiratory fitness); BW, body weight.

### Study Design

All testing was completed at the Translational Research Institute at AdventHealth, Orlando. Visit #1 consisted of a fasting blood draw, physical measurements, medical history/physical activity questionnaires, and resting electrocardiography (ECG). On visit #2, participants completed a VO_2_ max test with ECG to determine cardiorespiratory fitness. On visit #3, participants completed a dual energy X-ray absorptiometry (DXA) scan and were given a triaxial accelerometer to determine free living physical activity over a 1-week period. On visit #4, fasting blood samples were drawn into K2-EDTA tubes (pre-exercise), and participants then consumed a small low glycemic index meal (200 kcal, 15% protein, 35% fat, and 50% carbohydrate). Participants then completed the acute exercise bout on a cycle ergometer, as described in the next sections.

### Cardiorespiratory Testing and Assessment of Body Composition

VO_2_ max was determined as peak aerobic capacity measured during a graded exercise protocol on an electronically braked cycle ergometer, as previously described (Coen et al., 2013; Dube et al., 2014). Heart rate, blood pressure, and ECG (12-lead) were recorded throughout this test. The test was terminated according to the criteria outlined in the American College of Sports Medicine exercise testing guidelines (Thompson et al., 2010). DXA scans were performed using a GE Lunar iDXA whole-body scanner and analyzed with enCORE software. Coefficients of variation (CV) vary by tissue type and range from 0.5% to 1.1%. This CV data was provided by the manufacturer (for reference, see Faulkner et al., 2006).

### Daily Physical Activity

The monitor used for this study was the SenseWear^®^ Pro Armband (BodyMedia Inc., Pittsburgh, PA, United States). This activity monitor uses accelerometry, galvanic skin response, and skin temperature to estimate energy expenditure at a 1-min resolution (Berntsen et al., 2010). The SenseWear^®^ Armband has been demonstrated to be a valid, accurate, and reliable method when compared with indirect calorimetry (Berntsen et al., 2010) and doubly labeled water (Mackey et al., 2011). SenseWear professional version 8.1 software was used for data analysis. Participants were instructed to wear the armband on their upper left arm at all times over the seven-day period, with the exception of showering/bathing. Only days with a wear time of at least 85% were considered for further analysis.

### Acute Exercise Testing

Study participants performed an acute bout of aerobic exercise on a cycle ergometer as previously described (Distefano and Goodpaster, 2018). Following a 6-min warm-up, participants cycled for 40 min at ∼70% heart rate reserve, which was calculated during the maximum aerobic capacity test. Heart rate, perceived exertion, and blood pressure were measured every 5 min. Indirect calorimetry was examined with an open-circuit spirometry metabolic monitoring system (Parvo Medics, Sandy, UT), and used to verify exercise intensity. Blood was drawn before exercise (Pre), immediately after exercise (Post), and after 3 h of recovery (3hPost) via direct venipuncture using a 21-gauge butterfly needle, and blood samples were collected into PAXgene blood tubes with spray-coated K2-EDTA (Becton-Dickson, Catalog #366643).

### Plasma Isolation

Collected blood samples were processed according to the manufacturer’s instructions. Briefly, they were centrifuged at 3,000 ×*g* for 10 min at room temperature. Plasma was aliquoted and immediately stored at −80°C.

### Isolation and Enrichment of Exosomes From Plasma

Plasma was thawed on ice and centrifuged at 3,000 × *g* for 15 min at 4°C to remove particulate matter. Pre-cleared plasma (250 μl) was then mixed with an equal volume of thromboplastin D and incubated for 15 min at 37°C. Supernatants were collected following centrifugation at 13,000 × g for 10 min at 4°C, mixed with 100 μl of ExoQuick (System Biosciences, Palo Alto, CA, United States) and 6.25 μl of RNase (1 mg/ml), and incubated overnight at 4°C. Exosome-enriched preparations were spun down at 1,500 × *g* for 25 min at 4°C. Supernatants were then gently removed, and exosome-enriched pellets were resuspended in 50 μl of PBS with 1.25 μl of RNAse inhibitor (ThermoFisher Scientific, Waltham, MA, United States) and stored at −20°C.

### Nanoparticle Tracking Analysis

Exosome-enriched preparations were measured and quantified using a NanoSight NS300 instrument (Malvern Panalytical Ltd, Malvern, United Kingdom) equipped with a 532 nm laser and running NTA v3.2 software. These preparations were thawed on ice and gently resuspended immediately before sampling to prevent the settling of particles. Immediately before measurement, the exosome-enriched aggregates were broken up by gentle passage through a needle. Samples were diluted empirically with 0.22 mm filtered PBS to achieve 20–60 particles/frame (manufacturer’s optimum measurement range) using 1.5 ml low-adhesion tubes (United States Scientific, Orlando, FL, United States) to prevent exosome adsorption onto the walls of the tube. Diluted samples were loaded into the assembled sample chamber of a NanoSight NS300 instrument through a 1-ml syringe, applying each sample slowly to expel air without bubble formation. The chamber was then connected to the instrument and laser-engaged, and microparticles were brought into focus using the thumb print region as a reference; three 60-second video images/sample were acquired and analyzed using NanoSight NTA 2.3 software. Individual members of the prepared serial dilutions were analyzed until the detected raw concentration was within the recommended range for the instrument (1 × 10^8^ – 1 × 10^9^ particles/ml).

### RNA Isolation From Exosome-Enriched Preparations

For RNA isolation from exosome-enriched preparations, 700 μl of QIAzol lysis reagent (Qiagen, Germantown, MD, United States) were added to 50 μl of exosome preparation, and the mixture was incubated for 5 min at room temperature. After the addition of 140 μl of chloroform, the mixture was vortexed for 15 s and incubated for 3 min at room temperature. Following incubation, the mixture was centrifuged at 12,000 × *g* for 15 min at 4°C. After the upper aqueous phase was transferred to a fresh tube, a two-fold volume of absolute ethanol (Sigma-Aldrich, St. Louis, MO, United States) was added, and the mixture was vortexed briefly. Samples were then transferred to RNeasy Mini spin columns (Qiagen) that were centrifuged at 10,000 × *g* for 30 s at 4°C, and flow-through was discarded. An 80-μl volume of DNase incubation mix was added directly onto the column membrane and samples were incubated for 15 min at room temperature. Following wash steps with RWT buffer, RPE buffer (Qiagen), and 85% ethanol, RNA pellets were dried by centrifugation at 10,000 × *g* for 2 min at room temperature and eluted in 15 μl of water. Electrophoretic analysis of the RNA preparations was conducted on an Agilent 2100 Bioanalyzer using the Agilent Small RNA kit (Agilent, Santa Clara, CA, United States).

### Small RNA Library Preparation

Small RNA libraries were prepared using the NEBNext Small RNA Library Prep Set for Illumina (New England Biolabs, Ipswich, MA, United States), according to the manufacturer’s instructions. Briefly, RNA-Seq libraries were constructed using 2 ng of RNA isolated from exosome-enriched preparations and 15 cycles of PCR amplification. Libraries were run on a 6% TBE gel for 60 min at 120 V, and bands between 140 to 160 bp were excised from the gel using a razor blade. Gel pieces were crushed into smaller pieces and incubated in water overnight on a rotator. Libraries were isolated using gel filtration columns. Following ethanol precipitation, the resulting pellets were each resuspended in 11 μl of ultrapure water.

### Small RNA Library Sequencing and Data Analysis

All libraries were analyzed on an Agilent 2100 Bioanalyzer using the Agilent High Sensitivity DNA kit. For sequencing, libraries were pooled at equimolar concentrations, mixed with 5% PhiX (Illumina, San Diego, CA, United States), and single-end sequencing was performed on an Illumina MiSeq platform following the manufacturer’s recommendations. Sequencing data have been deposited to GEO (GSE144627).

Small RNA-seq reads were processed using the exceRpt pipeline (Rozowsky et al., 2019), as recommended by the ERC Consortium^1^. The pipeline initially trims the reads using FASTX-Toolkit to remove remnants of the 3′-adapter sequence followed by removal of the reads of low-quality and homopolymer repeats, and subsequently aligns the trimmed reads to NCBI’s Univec database to filter out contaminants. The good reads were mapped to rRNA, and then to the human genome and transcriptome to obtain the counts of different gencode and RNA transcripts including miRNA and tRNA. The order of annotations is as follows: miRNAs (miRBase v21), tRNAs, piRNAs, gencode transcripts (hg38 Gencode v24; snRNAs, snoRNAs, miscRNAs, protein coding mRNAs, and lncRNAs), and circular RNAs. Mapping of the reads to the NCBI’s Univec database or rRNA or human genome/transcriptome was performed using STAR 2.4.2a (Dobin et al., 2013).

### Statistics

Subject characteristics, physiological and exomiR data were reported as the mean ± standard error of the mean (SEM). For subject characteristics and physiological data, paired variables were compared using a Student’s *t*-test or a Wilcoxon signed-rank test as appropriate for the data distribution. When comparing miRNA expression changes either between time points (within the same group) or between groups (baseline), the Bioconductor DESeq2 package was used (Love et al., 2014). A log2 fold change ≥ 1 in either direction together with a Wald test *p*-value ≤ 0.05 indicated a statistically significant difference. Differentially expressed miRNAs were displayed in volcano plots and a heatmap. The computation was done in R 3.6.1 (R Core Team, 2019) and Bioconductor 3.10 (Gentleman et al., 2004) packages.

## Results

### Anthropometric and Physiological Characteristics of Participants

The study cohort (*n* = 10) comprised 5 older endurance-trained and 5 age-matched sedentary subjects, all of whom completed a 40 min bout of cycling exercise. Subject characteristics are provided in Table 1. The study groups were matched for age (all were male) and trained participants had significantly higher cardiorespiratory fitness (VO_2_ max), compared to the sedentary group, as per the study design. The sedentary group tended to have greater total fat mass and weight compared to the trained group. In contrast, trained and sedentary groups had comparable skeletal muscle index (SMI), and total leg lean mass.

Physiological characteristics measured during the acute exercise bout are presented in Table 2. While steady state VO_2_ and power output were significantly higher in the trained group, the participants performed the exercise bout at a similar relative intensity (% heart rate reserve (HRR) and % VO_2_ max). Additionally, the average respiratory quotient (RQ), an index of substrate metabolic utilization, was similar among the groups.

**TABLE 2:** Physiological data captured during acute exercise bout.

	**Trained (*n* = 5)**	**Sedentary (*n* = 5)**	T-test*
AVG HR (bpm)	119.6 ± 6.8	109.2 ± 7.8	0.347
%HRR (%)	68.8 ± 3.5	60.0 ± 7.2	0.189
SS VO_2_ (ml/kgBW/min)	22.1 ± 1.2	13.1 ± 1.3	< 0.001
SS %VO_2_ max (%)	63.9 ± 4.0	60.1 ± 3.9	0.605
AVG Power Output (Watts)	105.3 ± 5.6	45.8 ± 4.5	< 0.001
AVG RQ (VCO_2_ / VO_2_)	0.87 ± 0.01	0.85 ± 0.01	0.297

Data were recorded during the final 10 minutes of the exercise bout. Values are expressed as mean +/− SEM or mean percentage +/− SEM. *Unpaired *t* test; AVG, average; HR, heart rate; HRR, HR reserve = [maximum HR − resting HR]; SS VO_2_, VO_2_ during steady state exercise; RQ, respiratory quotient.

### Characterization of Exosome-Enriched Preparations

**FIGURE 1:**
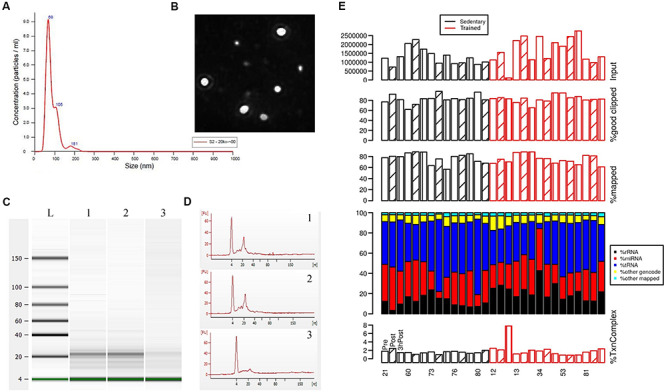
Characterization of exosome-enriched preparations isolated from plasma and analysis of their small RNA contents. **(A)** Analysis of EV size distribution and concentration in exosome-enriched preparations using a NanoSight NS300 instrument. **(B)** Representative image from the NanoSight instrument showing optimal light scatter from plasma-derived nanovesicles. **(C,D)** Bioanalyzer traces of the small RNAs extracted from exosome-enriched preparations following incubation with DNase or RNase. Lane 1, Control (non-treated); lane 2, DNase-treated; lane 3, RNase-treated. **(E)** Sequencing analysis of the small RNAs extracted from exosome-enriched preparations. Blood was drawn from each study participant at 3 time points: before exercise (Pre; open bars), immediately after exercise (Post; diagonal line bars), and 3 h after exercise (3 hPost; vertical line bars). Participants comprised groups of older trained and sedentary adults, as indicated. Shown at the top are the total number of reads, the percentage of mappable reads after adapter clipping, and the percentage of reads mapping to the human genome/transcriptome. Stacked-bar plots represent the percentage of mapped reads assigned to miRNA, rRNA, tRNA, other gencode transcripts (which are not miRNA, rRNA or tRNA), and other mapped sequences (mapped to the genome, but not to the four transcript types mentioned above). The different RNA species are color-coded. Shown at the bottom is the percentage of transcriptome complexity, defined as the ratio of the number of unique transcript sequences to the total number of transcript sequences.

We sought to validate our methodology for the isolation and enrichment of exosomes and for RNA extraction from the exosome-enriched preparations. Exosome-enriched preparations were isolated from blood plasma samples obtained from study participants, as described in the Materials and Methods. Analysis of exosome-enriched preparations using nanoparticle tracking analysis (NTA) allowed us to obtain the size distribution of EVs and an estimate of particle concentration with high resolution (Dragovic et al., 2011). Size distribution ranged from 68 to 105 nm, with a mean size of 85.1 nm (Figures 1A,B), which falls into the size range of exosomes (Jayaseelan, 2019), suggesting that the highly purified EVs contained mostly exosomes. Exosome concentrations in our preparations ranged from 5 × 10^8^ to 1 × 10^9^ particles/ml. We next examined RNA composition in the exosome-enriched preparations. Characterization of the RNA from exosome-enriched preparations by Bioanalyzer analysis revealed that the majority of RNA species (60–80%) were approximately 22 nt in size (Figures 1C,D). Those small RNA preparations were not degraded by DNase. However, they were completely degraded by RNase treatment (Figures 1C,D), thus demonstrating the integrity of small RNAs present in the exosome-enriched preparations.

### Characterization of Small RNAs Isolated From Exosome-Enriched Preparations

We performed sequencing of the small RNA libraries to characterize the circulating exosomal miRNAs from older trained and sedentary subjects and study their dynamic regulation following exercise. In our sequencing data, the percentage of mappable reads after adapter clipping ranged from 60 to over 90%. About 70–90% of total reads were mapped to the human genome and the transcriptome complexity was homogeneous across all samples. The QC of the sequencing data did not show bias towards either a group (trained vs. sedentary) or a time point. Overall, our small RNA sequencing data (Figure 1E) passed the raw read data QC when compared with the ERC Consortium QC standards for small RNA-seq data (see^2^).

Using mapped sequence read counts, we quantified the RNA contents of exosome-enriched preparations. As illustrated in Figure 1E, exosome-enriched preparations isolated from plasma contained a very diverse RNA ‘cargo’. As expected from previous research (Li et al., 2014; Perez-Boza et al., 2018), a large proportion of miRNAs, tRNAs, and rRNAs were detected, as well as a small fraction of other gencode transcripts including snRNAs, snoRNAs, miscRNAs, protein coding mRNAs, and lncRNAs. MicroRNAs were among the topmost abundant RNA species, accounting for 28.69 ± 8.76% of all mappable reads for all samples (all subjects and all time points). At the current read depth, 371 ± 71 miRNAs were detected. The five most common miRNAs (miR-99a-5p, miR-129-5p, miR-128-3p, let-7b-5p, miR-423-5p) collectively accounted for approximately 50% of all mappable miRNA sequences of all samples together. One of the most abundant miRNAs in red blood cells, miR-16 (Kirschner et al., 2011), was hardly detectable amongst all samples (about 2.63%), thus corroborating the lack of hemolysis during sample preparation. Altogether, our characterization of RNA content in exosome-enriched preparations is in agreement with previous reports (Li et al., 2014; Yeri et al., 2017) and supports the validity of our experimental approach for analyzing miRNAs in exosomes.

### Older Trained and Sedentary Subjects Show Different Circulating ExomiR Profiles at Baseline

**FIGURE 2:**
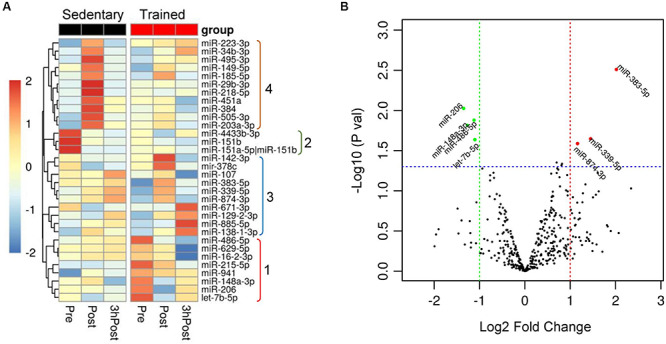
Differential expression of circulating exomiRs at baseline in the trained and sedentary groups. **(A)** Heat map with dendrogram illustrating clustering results for the 32 exomiRs identified as differentially expressed when comparing miRNA expression changes between groups (sedentary vs. trained) at baseline (Pre) and between time points (Post vs. Pre and 3hPost vs. Pre) within each group. Hierarchical clustering was applied using the average linkage method and Euclidean distance. Values signify the log2-transformed cpm (counts per million reads of the library size) normalized within each row such that the mean is zero and the standard deviation is one. Colors represent the level of miRNA expression; red: high expression; green: low expression. Groups numbered from 1 to 4 on the right highlight exomiRs that were differentially expressed at baseline (1 and 2) and at Post (4) between sedentary and trained subjects, and those differentially expressed at either Post or 3 hPost relative to Pre in trained subjects (3). **(B)** Differential expression of circulating exomiRs in the trained relative to sedentary group at baseline (Pre). The *p*-value in log10 scale of each miRNA is plotted against its fold change in log2 scale and each circle denotes a miRNA. The miRNAs with *p*-values ≤ 0.05 and log2 (fold change) ≥ 1 in either direction is represented by either red circles (upregulated) or green circles (downregulated). Three circulating exomiRs were upregulated and one was downregulated in the trained compared to sedentary group.

To assess similarities and differences in circulating exomiR profiles in the trained and sedentary groups, we employed a hierarchical clustering method comparing circulating exomiR profiles at baseline (Pre), immediately after acute exercise (Post), and 3 h after acute exercise (3hPost). We initially identified the miRNA species that were differentially expressed (i) at Pre between groups, (ii) between time points (Post vs. Pre, 3hPost vs. Pre, and 3hPost vs. Post) within each group. We then applied hierarchical clustering to these thirty-two differentially expressed miRNAs. As shown in Figure 2A, miRNA clusters in trained vs. sedentary subjects differed dramatically at baseline. For instance, miR-486-5p and miR-215-5p showed higher levels of basal expression in trained individuals than in the sedentary.

Comparison of the circulating exomiR profiles in trained and sedentary subjects at baseline revealed that four miRNA species were differentially expressed in the trained group (Figure 2B). Of those, three miRNAs (miR-486-5p, miR-215-5p, and miR-941) were upregulated, and one (miR-151b) was downregulated. Differential expression of muscle-enriched miR-486-5p was consistent with various studies reporting significant changes in miR-486-5p levels following exercise (Aoi et al., 2013; Denham and Prestes, 2016; D’Souza et al., 2017, 2018; Barber et al., 2019). Overall, these distinct patterns of miRNA expression indicate that regular exercise may be associated with significant and durable expression changes in resting plasma exomiRs.

### Acute Exercise Regulates Distinct Circulating ExomiRs in Trained and Sedentary Subjects

**FIGURE 3:**
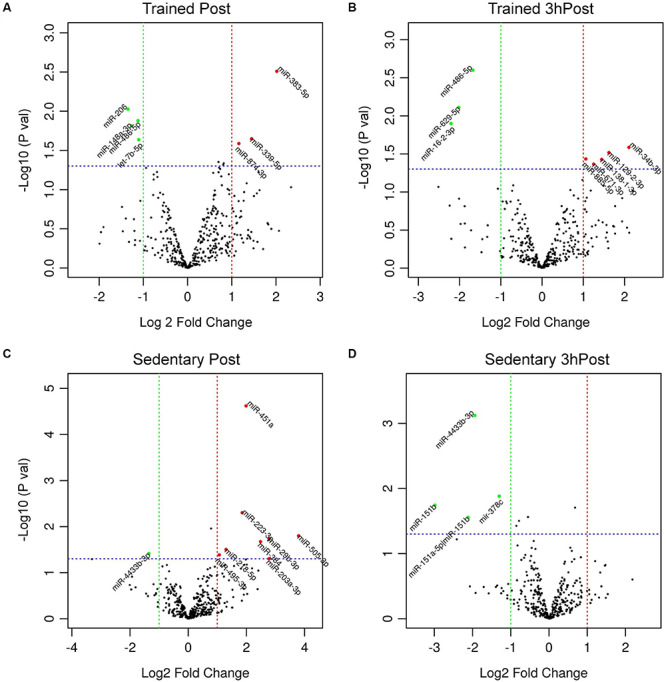
Differential regulation of circulating exomiRs following acute exercise in the trained and sedentary groups. **(A)** Differentially regulated circulating exomiRs immediately after acute exercise and 3 h post-exercise **(B)** compared to baseline (Pre) in trained group. **(C)** Differentially regulated circulating exomiRs immediately after exercise and 3 h post-exercise **(D)** compared to baseline in sedentary group. The *p*-value in log10 scale of each miRNA is plotted against its fold change in log2 scale and each circle symbolizes a miRNA. The miRNAs with *p*-values ≤ 0.05 and log2 (fold change) ≥ 1 in either direction are represented by either red circles (upregulated) or green circles (downregulated). In **(A)** three miRNAs were upregulated and four were downregulated. In **(B)** five miRNAs were upregulated and three were downregulated. In **(C)** eight miRNAs were upregulated and one was downregulated. In **(D)** all four miRNAs were downregulated.

In order to identify acute exercise-regulated miRNAs, we next compared post-exercise profiles (either Post or 3hPost) with the resting profile (Pre) within each group. In the trained group, we identified seven differentially regulated miRNAs at Post relative to Pre (Figures 2A, 3A). Among those, three miRNAs were upregulated (miR-383-5p, miR-339-5p, and miR-874-3p) and four were downregulated (miR-206, miR-486-5p, miR-148a-3p, and let-7b-5p). At 3hPost, eight exomiRs were differentially regulated (Figures 2A, 3B), five of which were upregulated (miR-34b-3p, miR-129-2-3p, miR-138-1-3p, miR-671-3p, and miR-885-5p), and three downregulated (miR-486-5p, miR-629-5p, and miR-16-2-3p). Remarkably, miR-486-5p was the only miRNA species to be shared by both post-exercise signatures. The levels of miR-486-5p in the trained group were significantly decreased at both Post and 3hPost, whereas at baseline miR-486-5p expression was higher than in the untrained (Figure 2B).

**FIGURE 4:**
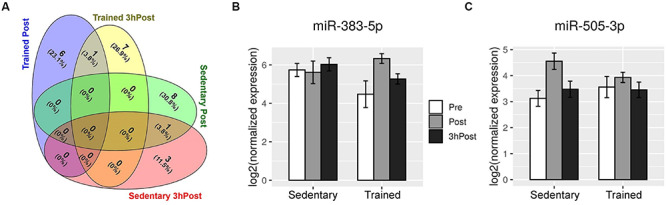
Acute exercise regulates distinct circulating exomiRs in the trained and sedentary groups. **(A)** Venn diagram depicting the exomiRs that were differentially regulated at Post and at 3 hPost in the trained group (7 and 8, respectively) and in the sedentary (9 and 4, respectively). There is no overlap between the trained and sedentary groups at either time point. **(B)** and **(C)** Shown are the expression levels of miR-383-5p **(B)** and miR-505-3p **(C)** in the circulating exosomes of sedentary and endurance-trained subjects before exercise (Pre), immediately after exercise (Post), and 3 h after exercise (3 hPost). Bar plots represent log2-transformed normalized read counts from small RNA sequencing.

Comparison of the Post and Pre exomiR profiles in the sedentary group identified nine differentially regulated exomiRs (Figures 2A, 3C). Except for miR-4433b-3p, all miRNAs (miR-505-3p, miR-29b-3p, miR-203a-3p, miR-384, miR-451a, miR-223-3p, miR-218-5p, and miR-495-3p) were upregulated in response to acute exercise. By contrast, all differentially regulated exomiRs at 3hPost (miR-4433b-3p, mir-378c, miR-151b, miR-151a-5p| hsa-miR-151b) were downregulated (Figures 2A, 3D). Except for miR-4433b-3p, there was no overlap between the two post-exercise signatures. Of note, miR-151b basal expression level was significantly greater in the sedentary compared to the trained group (Figures 2A, 3D). Overall, our data show that circulating exomiRs modulated by acute exercise in the trained and sedentary groups are totally distinct (Figure 4), and suggest that long-term exercise has a significant impact on the regulation of plasma exomiRs in response to acute exercise.

### Correlation of Exercise-Regulated ExomiRs With IGF1 Signaling

We employed pathway enrichment analysis (IPA), performed miRNA target prediction via miRDB (Wong and Wang, 2015), and mined the literature for experimentally validated miRNA targets to determine which molecular pathways would likely to be altered by the ExomiR signatures at baseline and in response to acute exercise. We found that the majority of differentially regulated miRNAs either target or are predicted to target components of the IGF1 signaling pathway. The IGF1 pathway, which signals through AKT-mediated activation of mTOR and repression of FOXO, and via the Ras/MAP kinase pathway, is a major regulator of muscle growth and glucose homeostasis (Schiaffino and Mammucari, 2011; O’Neill et al., 2015). IGF1 signaling is also implicated in exercise-induced muscle and cardiac hypertrophy (Velloso, 2008; Weeks and McMullen, 2011).

**FIGURE 5:**
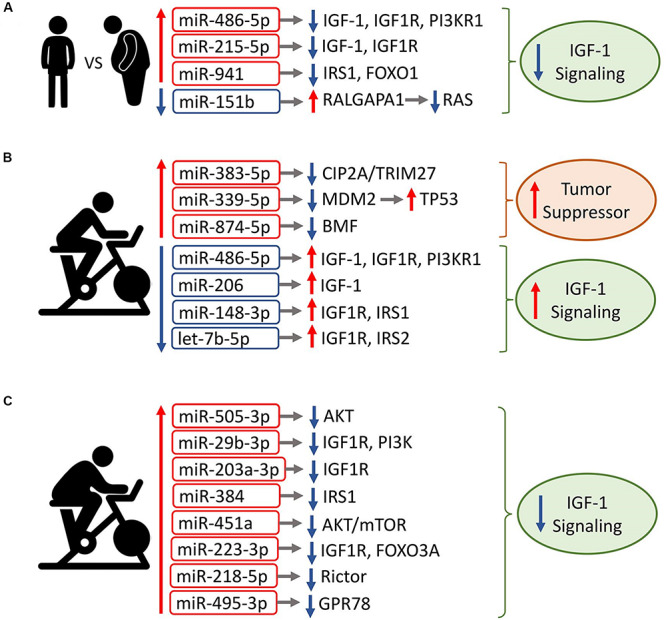
Association of circulating exomiR signatures with IGF1 signaling. Shown are the circulating exomiRs that were differentially regulated at Post, along with their reported/predicted target genes linked to IGF1 signaling, when applicable. Red border-rectangles indicate miRNAs that are upregulated; blue border-rectangles indicate miRNAs that are downregulated. Colors represent the level of miRNA expression; red: high expression; green: low expression. Up/down arrows indicate whether a target gene is up- or downregulated, respectively. **(A)** Circulating exomiRs that are differentially expressed in exercise-trained individuals relative to the sedentary lead to inhibition of IGF1 signaling. **(B)** In trained individuals, acute exercise-regulated exomiRs increase tumor suppressor activity and activate IGF1 signaling. **(C)** In sedentary individuals, the majority of acute exercise-regulated exomiRs are upregulated and result in downregulation of IGF1 signaling. Note that downregulated miR-4433b-3p is not shown because its target is undetermined.

Three of the four miRNAs in the trained subjects’ baseline exomiR signature (miR-486-5p, miR-215-5p, and miR-941) were previously shown to target components of the IGF1 signaling pathway (Pichiorri et al., 2010; Hu et al., 2012; Peng et al., 2013; Figure 5A). Additionally, downregulated miR-151b is predicted to target RALGAPA1, an inhibitor of Ras signaling (Yan and Theodorescu, 2018). Thus, the data suggest the hypothesis that resting exomiRs differentially modulate IGF-1 signaling in trained vs. untrained elderly subjects.

In the trained group’s Post vs. Pre signature, all three upregulated miRNAs (miR-383-5p, miR-339-5p, and miR-874-3p) have a tumor suppressive function (Zhang et al., 2014; Zhao et al., 2017; Jiang D. et al., 2019; Jiang J et al.,2019b; Figure 5B). In contrast, all four downregulated miRNA species target components of IGF1 signaling (Peng et al., 2013; Xu et al., 2013; Gao et al., 2014; Xu et al., 2014; Liu et al., 2018), suggesting that the exercise-induced exomiR signature in trained older adults activates IGF1 signaling. Except for miR-34b-3p (Hermeking, 2010), the majority of differentially regulated miRNAs at 3hPost vs. Pre target components of IGF1 signaling (Peng et al., 2013; Ye et al., 2015; Meier et al., 2016; Ntoumou et al., 2017; Yan et al., 2017; Zhu et al., 2018; Han et al., 2019), suggesting that they may also modulate the IGF1 pathway.

In contrast with the trained group, all but one miRNA species were upregulated in the sedentary’s Post vs. Pre signature. Downregulated miR-4433b-3p has no established or predicted gene target related to IGF1 signaling. The majority of increased miRNAs (seven out of eight) repress components of IGF1 signaling (Uesugi et al., 2011; Olivieri et al., 2014a; Yuan et al., 2015; Kim et al., 2016; Lai et al., 2016; Minna et al., 2016; Li et al., 2017; Chen et al., 2018; Wang Z. et al., 2018; Tang et al., 2019; Zeng et al., 2019), suggesting that the exercise-induced exomiR signature in the sedentary inhibits IGF1 signaling (Figure 5C). The majority of differentially regulated miRNAs at 3hPost vs. Pre either target or are predicted to target components of IGF1 signaling (Knezevic et al., 2012), implying a modulation of this pathway. Collectively, our findings suggest that exercise-regulated plasma exomiRs modulate IGF1 signaling. Particularly, the plasma exomiR signatures induced immediately after exercise may have opposing effects on IGF1 signaling in trained vs. sedentary aging adults.

## Discussion

In the present study, we examined for the first time the effects of chronic and acute endurance exercise on global circulating exomiR profiles in older adults, using a next-generation sequencing-based approach. We identified a subset of differentially expressed plasma-derived exomiRs that target genes the IGF-1 signaling pathway in a long-term exercise (trained) compared to an untrained (sedentary) older subjects. We also found that acute exercise-induced plasma exomiR signatures in trained and untrained groups are distinct and have opposing effects on IGF-1 signaling. IGF-1 signaling is an important regulator of muscle growth and glucose homeostasis (Schiaffino and Mammucari, 2011; O’Neill et al., 2015; Vassilakos et al., 2019). In light of the known relationship between exercise and IGF-1 signaling (Velloso, 2008) and the dysregulation of IGF-1 signaling associated with aging (Rebelo-Marques et al., 2018), exercise-regulated circulating exomiRs might contribute to a differential regulation of IGF-1 signaling in trained compared to sedentary elderly subjects, suggesting their role as mediators of exercise-induced adaptations in the elderly population. Thus, our data could provide novel insights into the molecular mechanisms underlying the physiology of exercise in the elderly population.

Among the species regulated with exercise, we identified two muscle-enriched miRNAs (Horak et al., 2016), miR-486-5p and miR-206, whose exosomal levels were downregulated in trained individuals immediately after a single bout of acute exercise. Additionally, miR-486 baseline level was greater in the older trained group than in the sedentary group (Figure 2A). Previous studies have shown that miR-486 was more abundant in resting athletes’ whole blood than in healthy controls, whereas it was decreased following acute exercise in healthy controls (Denham and Prestes, 2016). In skeletal muscle, miR-206 was unaltered following acute exercise, but significantly decreased following 12 weeks of endurance training (Nielsen et al., 2010). Validated targets of miR-486 include both positive (IGF-1, IGF-1R, PIK3R1) and negative (PTEN) components of IGF-1 signaling (Small et al., 2010; Peng et al., 2013), suggesting that miR-486 may have both activating and inhibiting effects on PI3K/AKT signaling. Because activation of IGF-1/PI3K/AKT signaling is recognized to promote skeletal and cardiac muscle growth (Antos et al., 2002; Stitt et al., 2004; Vassilakos and Barton, 2018), we anticipate miR-486 to be involved in the regulation of muscle growth. MiR-206, which inhibits the PI3K/AKT signaling pathway in rat (Liu et al., 2018), was previously shown to promote skeletal muscle differentiation (Kim et al., 2006). Overall, the presence of these muscle enriched miRNAs in post-exercise signature is consistent with the impact of exercise on skeletal muscle hypertrophy and regeneration in response to long-term regular exercise (Bazgir et al., 2017).

In addition to muscle-enriched miRNAs, we found various non-muscle-specific miRNAs in the plasma exomiR profiles of elderly subjects following exercise. Some of these miRNA species, e.g., miR-215-5p, miR-941, miR-671-3p, miR-203a-3p, miR-4433b, and miR-384, have not been linked to exercise thus far, while others, like miR-29b, miR-451, miR-223, miR-505, and miR-629 have. Modulation of miR-29b and miR-451 following exercise training was formerly reported in skeletal muscle (Keller et al., 2011; Russell et al., 2013), and miR-29b was shown to promote muscle atrophy via the targeting of IGF-1 and PI3K (Li et al., 2017). MiR-451a also targets the AKT/mTOR pathway (Minna et al., 2016; Olioso et al., 2019), although its inhibitory effect on myogenesis in mice is not AKT-mediated (Munk et al., 2019). Our observations of exercise-induced alterations in exosomal miR-29b and miR-451 are consistent with previous studies and suggest that they could be involved in the regulation of skeletal muscle adaptations to exercise. Remarkably, upregulation of miR-29b following exercise was previously demonstrated in heart tissue-derived exosomes, suggesting a potential role in cardiac remodeling (Chaturvedi et al., 2015). Exercise-induced alterations in miR-223, miR-505, miR-629, and miR-486 expression were reported in immune cells (blood neutrophils, natural killers, and PBMCs), supporting their role as potential mediators of the effects of acute exercise on the immune system and inflammatory mechanisms (Radom-Aizik et al., 2010, 2012, 2013; Simpson et al., 2012; Nieman and Wentz, 2019). Both miR-223 and miR-629 were shown to target FOXO3A, a key mediator of the PI3K/AKT signaling pathway (Olivieri et al., 2014a; Kim et al., 2016; Yan et al., 2017), while miR-505 was recently shown to inhibit AKT phosphorylation (Tang et al., 2019). Collectively, our data support the concept that circulating exomiRs may be released by various tissues besides skeletal muscle, thereby contributing to the multisystemic benefits of exercise (for review, see Safdar and Tarnopolsky, 2018).

There is a paucity of data on the regulation of circulating exomiR expression by exercise (Guescini et al., 2015; D’Souza et al., 2018; Lovett et al., 2018; Hou et al., 2019). Inversely, numerous studies have reported the modulation of cell-free circulating miRNAs following exercise (Baggish et al., 2011; Aoi et al., 2013; Mooren et al., 2014; Nielsen et al., 2014; D’Souza et al., 2017; Shah et al., 2017; Horak et al., 2018; Barber et al., 2019). In the present work, older trained individuals displayed significant changes in exosomal miR-383-5p, miR-874-3p, miR-206, miR-486, miR-148, and let-7b levels immediately after exercise. Previous studies on circulating miRNAs reported significant expression changes in these six miRNA species following acute or chronic aerobic exercise (Nielsen et al., 2010; Aoi et al., 2013; Mooren et al., 2014; Nielsen et al., 2014; Denham and Prestes, 2016; D’Souza et al., 2017, 2018; Barber et al., 2019; Faraldi et al., 2019; Tung et al., 2019), thus strengthening our findings. Comparison of our exercise-induced plasma exomiR alterations with those from previous studies on circulating exosomes revealed both similarities and differences. Similar to the D’Souza study (D’Souza et al., 2018), we found an increase in miR-451a, a miRNA expressed in various tissues (Ludwig et al., 2016), in sedentary individuals following exercise. However, while we observed a decrease in muscle-specific miR-486-5p in trained individuals post-exercise (at both Post and 3hPost), other groups reported either an increase (D’Souza et al., 2018) or no significant change in untrained subjects (Lovett et al., 2018). Contrary to a recent study (Hou et al., 2019), we saw no significant change in baseline miR-342-5p in trained individuals. Since all of those previous studies involved young men, differences in the age of the cohort and in exercise regimens may likely contribute to these variations.

The mechanisms involved in exosome formation and secretion are poorly understood (Stoorvogel, 2012). Exercise-induced downregulation of miR-486 and miR-206 in older trained individuals might reflect an intake by cardiac or skeletal muscle cells, as these species are enriched in cardiac and skeletal muscle (Small et al., 2010; Kirby and McCarthy, 2013). By contrast, the higher miR-486 levels observed in the trained group at baseline could reflect an increase in muscle miRNA export, and/or either an increase in miRNA biogenesis or a decrease in its degradation. Acute exercise was previously shown to cause a transient increase in the level of circulating exosomes (Fruhbeis et al., 2015; Lovett et al., 2018; Whitham et al., 2018). Although variation in particle number or size was not evaluated in this study, we anticipate that there be a similar increase in EV levels immediately after exercise. Additionally, it would be important to determine whether the increase in exomiRs is due to an increase in a subpopulation of exosomes containing these specific miRNAs, or/and to increased packaging of these miRNAs. New technologies are being developed to analyze individual vesicles and identify heterogeneous EV populations (in terms of size and composition) and EV subtypes (Hartjes et al., 2019). Thus, further investigation is needed to elucidate the molecular basis of miRNA transfer by exosomes and determine the kinetics of miRNA turnover and the functional roles of differentially regulated exomiRs.

The differential expression of resting plasma-derived exomiR in older trained relative to the sedentary subjects suggests that exercise training exerts long-term alterations on circulating exomiR profiles. This idea is supported by a recent study by Hou et al. demonstrating increased levels of a circulating exomiR (miR-342-5p) in young athletes compared with untrained students (Hou et al., 2019). Similarly, the levels of circulating miRNAs and intramuscular muscle-specific miRNAs are altered following exercise training (Nielsen et al., 2010, 2014; Baggish et al., 2011; Aoi et al., 2013; Horak et al., 2016; Barber et al., 2019), and pronounced intramuscular gene expression changes have been described following two months of endurance training (Schmutz et al., 2006; Popov et al., 2019). Hence, in older adults, circulating exomiRs that are induced by exercise training could mediate some of the long-term physiological adaptations in muscle, heart, and other tissues (Rivera-Brown and Frontera, 2012), conceivably via regulation of IGF-1 signaling.

Exercise stimulates IGF-1 synthesis directly in muscle, and indirectly in most tissues via the stimulation of growth hormone secretion (Velloso, 2008). In muscle, both exercise and IGF-1 activate the AKT/mTOR signaling pathway, which stimulates protein synthesis and results in muscle hypertrophy. In the heart, exercise-induced cardiac hypertrophy is dependent on IGF-1 signaling (Troncoso et al., 2014; Wang L. et al., 2018). The divergent effects of plasma exomiR signatures on IGF-1 signaling (both at pre and post) in trained compared to untrained older subjects echo previous reports showing: (i) a higher anabolic response (greater muscle mass) and higher insulin sensitivity in endurance-trained old individuals compared to the untrained (Mikkelsen et al., 2013), and (ii) an activation of IGF-1 signaling in senescent rats subjected to acceleration training (based on muscle protein quantification) (Launay et al., 2017). As aging is associated with reduced IGF-1 levels and decreased IGF-1 signaling (Rebelo-Marques et al., 2018), it is tempting to speculate that regular exercise induces plasma exomiR changes that may help to counteract age-related anabolic resistance. Further investigation will be needed to ascertain this hypothesis, including measurements of IGF-1 levels in blood and/or IGF-1 signaling components in skeletal muscle biopsies, and an evaluation of the effect of regular exercise in a group of untrained elderly subjects (e.g., before and after completing 20 weeks of endurance training).

Identification of several regulated miRNAs that have been reported in other studies provides support for the validity and reproducibility of our findings. However, we note that our study is limited in cohort size, and that replication and extension of our findings in a larger cohort would be valuable. We acknowledge that without a non-exercise control group the study design does not control for the effect of dietary status. Furthermore, our study is limited to males. Given the differences between men and women with regard to the physiological response to exercise (Sheel, 2016), it would be interesting to evaluate the impact of exercise training and acute exercise on plasma exomiRs in older females as well.

We also note methodological limitations in our study. Our EV characterization lacks the characterization of EV protein contents (e.g., by Western blot) as well as the visualization of single EVs using high resolution electron microscopy (e.g., by transmission electron microscopy) (for reference, see Thery et al., 2018). While the ExoQuick precipitation-based method used for exosome isolation we used elicits a lower protein purity compared to other methods (Tang et al., 2017; Macias et al., 2019), it shows a higher particle recovery, which is most appropriate for downstream analysis exosomal miRNAs. We used a low-bias protocol for small RNA library preparation. However, some sequence bias may still occur during adapter ligation (Baran-Gale et al., 2015; Dard-Dascot et al., 2018). We did not confirm our RNA-seq-based results with qPCR measurement of regulated transcripts, although others have reported good correspondence of miRNA levels measured by sequencing and by qPCR (Mitchell et al., 2018).

Beyond the potential utility of plasma-derived exomiRs as biomarkers of physical fitness and exercise physiology (e.g., muscle contractile activity), our work suggests their important role in the regulation of IGF-1 signaling in other tissues. Potential therapeutic applications include the assessment of exercise capacity in specific groups (e.g., overweight people), and predicting the risk for cardiovascular diseases (Gomes et al., 2015). Furthermore, this study sets the stage for a deeper inquiry into the dynamic biology of circulating exomiRs in response to exercise in the aging population.

## Data Availability Statement

The data that support the findings of this study are available from the corresponding author upon request.

## Ethics Statement

The studies involving human participants were reviewed and approved by Florida Hospital, AdventHealth Orlando (Orlando, FL) Institutional Review Board (IRBNet #554559). The patients/participants provided their written informed consent to participate in this study.

## Author Contributions

PC, VN, and SS designed the study. PC and BG recruited participants, collected samples, and performed experiments. VN, YG, SL, NJ, NS, MA, and MW performed experiments, collected and analyzed data, and revised the final version of the manuscript. VN and HP analyzed and interpreted data and drafted the manuscript. VN, HP, YG, PC, and SS critically revised the manuscript. All authors read and approved the final version of the manuscript.

## Conflict of Interest

BG and PC were employed by the company AdventHealth. The remaining authors declare that the research was conducted in the absence of any commercial or financial relationships that could be construed as a potential conflict of interest.

## Footnotes

1 https://exrna.org

2 https://exrna.org/resources/data/data-quality-control-standards/
